# Single-cell RNA-sequencing analysis of aortic valve interstitial cells demonstrates the regulation of integrin signaling by nitric oxide

**DOI:** 10.3389/fcvm.2022.742850

**Published:** 2022-10-25

**Authors:** Uddalak Majumdar, Talita Z. Choudhury, Sathiyanarayanan Manivannan, Yukie Ueyama, Madhumita Basu, Vidu Garg

**Affiliations:** ^1^Center for Cardiovascular Research, Nationwide Children’s Hospital, Columbus, OH, United States; ^2^The Heart Center, Nationwide Children’s Hospital, Columbus, OH, United States; ^3^Department of Pediatrics, The Ohio State University, Columbus, OH, United States; ^4^Department of Molecular Genetics, The Ohio State University, Columbus, OH, United States

**Keywords:** calcific aortic valve disease, nitric oxide, S-nitrosylation, single cell RNA-sequencing, integrin signaling, extracellular matrix (ECM)

## Abstract

Calcific aortic valve disease (CAVD) is an increasingly prevalent condition among the elderly population that is associated with significant morbidity and mortality. Insufficient understanding of the underlying disease mechanisms has hindered the development of pharmacologic therapies for CAVD. Recently, we described nitric oxide (NO) mediated S-nitrosylation as a novel mechanism for preventing the calcific process. We demonstrated that NO donor or an S-nitrosylating agent, S-nitrosoglutathione (GSNO), inhibits spontaneous calcification in porcine aortic valve interstitial cells (pAVICs) and this was supported by single-cell RNA sequencing (scRNAseq) that demonstrated NO donor and GSNO inhibited myofibroblast activation of pAVICs. Here, we investigated novel signaling pathways that are critical for the calcification of pAVICs that are altered by NO and GSNO by performing an in-depth analysis of the scRNA-seq dataset. Transcriptomic analysis revealed 1,247 differentially expressed genes in pAVICs after NO donor or GSNO treatment compared to untreated cells. Pathway-based analysis of the differentially expressed genes revealed an overrepresentation of the integrin signaling pathway, along with the Rho GTPase, Wnt, TGF-β, and p53 signaling pathways. We demonstrate that *ITGA8* and *VCL*, two of the identified genes from the integrin signaling pathway, which are known to regulate cell-extracellular matrix (ECM) communication and focal adhesion, were upregulated in both *in vitro* and *in vivo* calcific conditions. Reduced expression of these genes after treatment with NO donor suggests that NO inhibits calcification by targeting myofibroblast adhesion and ECM remodeling. In addition, withdrawal of NO donor after 3 days of exposure revealed that NO-mediated transcriptional and translational regulation is a transient event and requires continuous NO exposure to inhibit calcification. Overall, our data suggest that NO and S-nitrosylation regulate the integrin signaling pathway to maintain healthy cell-ECM interaction and prevent CAVD.

## Introduction

Calcific aortic valve disease (CAVD) is defined by the thickening of the aortic valve leaflets with the deposition of calcific nodules. The estimated global prevalence of CAVD is 12.6 million with greater than 100,000 deaths per year (1). The Global Burden of Disease Study 2019 found that newly diagnosed cases of CAVD increased ∼3.5-fold from 1990, with nearly 600,000 cases in 2019. (2). Despite its correlation with aging, calcification has been demonstrated to be an active disease process and not a degenerative process (3). However, the mechanisms underlying disease progression are not clearly understood and as a result, no effective therapies have been developed to prevent or cure CAVD in humans (4, 5).

A healthy aortic valve opens and closes to its full extent during each cardiac cycle to maintain proper unidirectional blood flow and prevent regurgitation. The structure and composition of the leaflet allow the valve to withstand the oscillating hemodynamic forces associated with each cardiac contraction. Heart valves are composed of valve endothelial cells (VECs), valve interstitial cells (VICs), and extracellular matrix (ECM). VICs are interspersed within the ECM, and the VICs and ECM are encapsulated by a single layer of VECs. Bidirectional communication between VECs, VICs, and ECM is important for the maintenance of a healthy valve. The present and previous studies have demonstrated that VEC-derived nitric oxide (NO), a second messenger molecule, is important for the inhibition of calcification in VICs (6–9). VECs modulate the proliferation and differentiation of VICs to maintain a quiescent VIC phenotype by reducing the expression of α-smooth muscle actin (αSMA), while the osteogenic phenotype of VICs is defined by the increased expression of RUNX2 (9, 10). Studies have shown that porcine aortic valve interstitial cells (pAVICs) when cultured on stiff surfaces express increased αSMA, which can be inhibited by co-culturing with porcine aortic valve endothelial cells (pAVECs) (11). The addition of L-NAME, a NO synthase (NOS) blocker, abrogates this VEC-dependent inhibition of αSMA expression (11). Regulation by VECs is dependent on blood flow and associated shear stress, which can also regulate NO production (12, 13). Exposure to L-arginine, a NOS substrate, can prevent lipopolysaccharide (LPS)-induced bone-related alkaline phosphatase expression and calcification of collagen matrix in bovine aortic valve interstitial cells (AVICs) (14). Recently, we observed NO donor can suppress both αSMA and RUNX2 expression in pAVICs (7). *In vivo* and *in vitro* studies have demonstrated that NO can activate NOTCH1 signaling, and loss of function mutations in *NOTCH1* are associated with CAVD in humans (6, 15). We have also demonstrated aortic valve thickening in *Notch1* haploinsufficient mice in a *Nos3^–/–^* background (6, 16). Recently, we described a mechanism by which NO activates NOTCH1 signaling and prevents CAVD that requires S-nitrosylation, a NO-dependent post-translational modification of target proteins (7). Utilizing mass spectrometric screening, we identified USP9X, a deubiquitinase, as one of the S-nitrosylated targets that can activate NOTCH1 signaling. We further demonstrated that the deletion of *Usp9x* from VECs and VEC-derived VICs in mice leads to thickened, calcified aortic valves which are stenotic (7). Although the involvement of the NO-dependent sGC/cGMP pathway in the inhibition of calcification of VICs has been described by others, we did not observe activation of this pathway in pAVICs culture condition after NO donor treatment (9, 17–19).

To understand the molecular mechanism of NO-dependent inhibition of CAVD, we utilized *in vitro* pAVICs (6). We observed spontaneous calcification of pAVICs when cultured on the stiff surface of tissue culture plastic plates. This calcification was prevented when cultured in presence of NO donor and the S-nitrosylating agent, S-nitrosoglutathione (GSNO) (7). Therefore, the pAVICs, cultured in presence of NO donor or GSNO represent a healthy state, whereas, the untreated cells represent a disease condition. It has been previously reported that the myofibroblast activation in pAVICs is initiated by increasing the stiffness of the culture conditions (20, 21). We demonstrated NO donor and GSNO can inhibit this stiffness associated myofibroblast activation and subsequent calcification. VICs are surrounded by ECM *in vivo*, and the ECM functions to maintain the proper healthy environment for VICs. We previously reported the presence of disorganized ECM with increased deposition of proteoglycan in *Usp9x* mutant mice, an *in vivo* model of CAVD (7). However, the role of NO in the regulation of ECM is not well-understood.

Communication between the valve cells and ECM is critical for the optimal function of the valve. In this context, cell surface receptors play critical roles in maintaining ECM homeostasis and reduced stiffness. Healthy VICs are responsible for the turnover of ECM and respond to extracellular mechanical and chemical stimuli (22). VICs communicate with the ECM through integrins and non-integrin membrane receptors, which play a regulatory role in stiffness-dependent calcification (20, 23). Integrins are transmembrane proteins comprising heterodimers of α and β subunits. Dysregulation in this integrin specificity has been observed in CAVD along with multiple other diseases including cancer, atherosclerosis, and kidney, and liver diseases (24, 25). Integrin receptors bind to their specific ECM partner proteins by recognizing distinct peptide sequences and blocking of this interaction has been shown to lead to calcific nodule formation (19). This suggests that the ECM-influenced calcification may be regulated by integrin-ECM interactions (20). For example, integrin α5β1 and αvβ3, which are both minimally expressed in quiescent VICs, bind to fibronectin by recognizing RGD peptide motif, but with myofibroblast activation, the expression and ECM binding of both of these receptors is increased (20, 25, 26). On the other hand, the 67-kD non-integrin protein receptor interacts with the laminin-derived YIGSR peptide motif. Blocking of this interaction leads to increase calcific nodule formation in pAVICs, suggesting its inhibitory role in calcification (20). Other integrin receptors, such as α2β1 bind to collagen-derived DEGA peptide motif, important for force generation in the valve leaflets (27, 28). Blocking of α2β1 integrins or actin polymerization abolished force generation that suggests VIC-collagen coupling *via* α2β1 is necessary for force generation in the valve leaflet (28). In addition, elevated expression of other collagen-binding integrin receptors α1β1 and α3β1 were also observed in diseased valves (25, 29). Certain integrin-ligand interactions play important role in the initiation of osteoblast differentiation and mineralization (30). This interaction between ECM ligands and integrin receptors initiates downstream signaling pathways by activating Ras or MAPK (31). After binding to ECM ligands, integrin receptors transmit extracellular mechano-signals inside the cells by recruiting focal adhesion proteins, which establish the connection to the actin cytoskeleton. In addition, α-smooth muscle actin (αSMA; *ACTA2*), expressed in myofibroblasts allows cells to generate enhanced cellular forces, which can also be transmitted back to the ECM *via* integrins (32). These mechanical forces prompt VICs to be activated and to differentiate into myofibroblasts, which then secrete growth factors and also ECM components. The role of transforming growth factors (TGF) has been implicated in valve development and calcification (33–37). Our published analysis of single-cell RNA sequencing (scRNAseq) data from pAVICs demonstrates that NO donor and GSNO prevent the myofibroblast activation and calcification by downregulating αSMA, Vimentin, Calponin, and Transgelin expression (7). However, our previous analysis was limited to the examination of genes that are important for myofibroblast activation. We did not examine the altered cellular signaling pathways, which are modulated by NO donor or GSNO to inhibit calcification.

In this report, we have analyzed scRNAseq data to identify the altered cellular signaling pathways that prevent spontaneous myofibroblast activation and calcification of pAVICs in response to NO signaling. We found an overrepresentation of the integrin signaling pathway in NO donor and GSNO-treated conditions. This finding was further confirmed in our recently described murine model of calcification. We also have demonstrated that altered gene expression in pAVICs after NO donor and GSNO treatment is temporary and is dependent on continuous NO exposure. Overall, our data suggest that NO and S-nitrosylation of target proteins maintain healthy VIC-ECM interactions and prevent calcification by regulation of integrin signaling pathway members.

## Materials and methods

### Porcine aortic valve interstitial cell culture and treatments

Valve interstitial cells were collected from juvenile pig valve leaflets as previously described (7). Briefly, valve leaflets were digested with collagenase (Worthington Biochemical# LS004176) for 5°min at 37^°^C and the VEC layer was removed gently with a sterile swab. After VEC layer removal, valve leaflets were digested with collagenase for 15 h at 37^°^C to dislodge VICs. Isolated pAVICs were cultured in a VIC-specific medium as previously described (7). pAVICs were passaged with trypsin-EDTA and only cells between passages three and seven were used in this study. pAVICs were cultured on a stiff surface of plastic tissue culture plates in Media-199 (ThermoFisher, Waltham, MA, United States, #11150059) supplemented with 10% fetal bovine serum (FBS) for 5 days or as otherwise noted. To expose pAVICs to the NO donor or S-nitrosylating agent, cell culture media was supplemented with either detaNONOate (150 μm) (FisherScientific, Pittsburgh, PA, United States, #AC328651000), or S-nitrosoglutathione (GSNO: 200 μm) (MilliporeSigma, Burlington, MA, United States, #N4148) and was refreshed daily.

To test and validate whether the effect of NO is long-lasting, pAVICs were cultured for 3 days with detaNONOate (150 μm) and then without detaNONOate either for additional 2 days (scRNAseq) or an additional 2, 5, and 8 days (Western Blot). This pAVICs culture condition will be designated as “NO donor withdrawal.” In this experiment 5 days culture of pAVICs with and without detaNONOate, and GSNO were utilized as control.

To culture pAVICs in osteogenic media, Media 199 was supplemented with ascorbate-2-phosphate (50 μg/ml; Sigma-Aldrich, Burlington, MA, United States, #49752), 10 nM dexamethasone (Sigma-Aldrich, Burlington, MA, United States, #D4902), and 10 μm β-glycerol phosphate (Sigma-Aldrich, Burlington, MA, United States, #G9422).

### Single-cell RNA sequencing and analyses

For this study, we examined published single-cell data (GEO accession no: GSE161123). The methods for single-cell sequencing were already described previously (7). Briefly, we utilized >85% viable pAVICs to generate 10 × Genomics 3′ v2 chemistry-based libraries using 10 × Genomics Chromium controller (∼2,000 target cell recovery/group) following the manufacturer’s instructions. All libraries were sequenced (150 bp paired-end) in the Illumina HiSeq4000 platform and 10× Genomics’ Cell Ranger pipeline was used to demultiplex and generate FASTQ files. Data was mapped to the pig genome Sscrofa11 with Y sequences from WTSI_X_Y_pig V2 (GCF_000003025.6_Sscrofa11.1_genomic.fna) and gene annotation (GCF_000003025.6_Sscrofa11.1_genomic_genes. filtered.gtf) from NCBI.^1^ Cell ranger aggregate was used to combine scRNA-seq data with different treatments (control, GSNO, NO donor, and NO donor withdrawal) that yield a total of 7,037 cells (control: 1,683, GSNO: 1966, NO donor: 1,485, and NO donor withdrawal: 1,903 cells). We recovered an average expression of 3,397 genes per cell. Seurat (version 3.0) was used for data processing, identification, and normalization of genes with the highest variability. Common differentially expressed genes among NO donor and GSNO compared to control were selected using criteria *p*_adjusted_ ≤ 0.05 and Log_2_FC ≥ 0.6 or ≤–0.6 (at least 50% up or down-regulated genes). For all of the samples, the base means cut-off of 100 was utilized to filter differentially expressed genes. The volcano plot was created using ggplot2 (3.2.1) package of R. Heatmap and violin plots were created using Ryabhatta App (38). The chord diagram was generated using http://www.datasmith.org online tool. PANTHER pathway analysis was performed using the functional classification of *Homo sapiens* PANTHER 16.0 release. In addition, we imported mouse (P30) aortic valve data (39) from the Gene Expression Omnibus database (GSE117011). To compare the gene expression of mouse aortic valve and pAVICs, mouse gene names were replaced with corresponding porcine homolog’s gene names downloaded from BioMart (Ensemble^2^) as previously described (7). To compare the trends in the gene expression change across different treatments, a normalized, average expression from the four treatments was used. Normalized, average expression values for each of the 1,247 genes were calculated using the “AverageExpression” function of Seurat. Spearman distances were calculated for each of the pairs of culture conditions using the “Dist” function in the R package “amap” (version 0.8.18). Heatmap was drawn using the R package Pheatmap (version 1.0.12) based on the distance matrix output of the Dist function.

### RNA purification and quantitative real-time polymerase chain reaction

RNA was isolated from pAVICs using TRIzol Reagent (ThermoFisher Scientific, Waltham, MA, United States, #15596018) following the manufacturer’s protocol. Approximately 0.5–1.0 μg of RNA was used for reverse transcription using SuperScript VILO cDNA Synthesis Kit (ThermoFisher Scientific, Waltham, MA, United States, #11754-050). For the quantification of gene expression, real-time PCR was performed using the Applied Biosystems 7,500 real-time polymerase chain reaction (PCR). SYBR Green-based (Thermo Fisher Scientific# 4385612) quantitative real-time (RT) PCR was performed to calculate mean relative gene expression after normalization of C_*t*_ values to glyceraldehyde 3-phosphate dehydrogenase (*GAPDH*) using the ^ΔΔ^ Ct method. *GAPDH* has previously been utilized as a control gene for normalization in aortic valve leaflets (40). All primer sequences are provided in Supplementary Data 2.

### Experimental mouse model

Animal experiments using mice were approved by the Institutional Animal Care and Use Committee (IACUC) at the Research Institute at Nationwide Children’s Hospital. *Usp9x^fl/fl^* mice were obtained from Charles River Laboratories, Italy, originally generated by Ozgene Pty Ltd., Australia. *Usp9x^fl/fl^* female mice were bred with *Tie2^Cre^* male mice (Jax# 008863), to generate *Cre*^+^ (experimental) and *Cre*^–^ (control) male (*Usp9x*^fl/Y^*; Tie2*^Cre^**) mice as described previously (7). Genotyping was performed at P10 for *Cre*. After 6 weeks, mice were fed a high-fat western diet (Envigo, Indianapolis, IN, United States, #TD.88137) until 24 weeks of age. Prior to euthanasia, female mice underwent echocardiograms following our previously described protocol (7). Only male mice were utilized for subsequent studies. Mice were euthanized to collect hearts for immunohistological analyses. After euthanasia, hearts were perfused with phosphate-buffered saline (PBS), removed, and fixed in 4% paraformaldehyde (PFA) at 4^°^C overnight. After fixation, the hearts were embedded in paraffin and sectioned for further analysis.

### Immunofluorescence and immunohistochemistry *in vitro* and *in vivo*

For immunofluorescence staining, cultured pAVICs were fixed with 2.5% PFA at 4°C for 15 min and permeabilized with Phosphate-Buffered Saline Triton (PBST, PBS containing 0.1% TritonX100). After permeabilization, non-specific immunoreactions were blocked using 1% BSA in PBST for 1 h at room temperature and incubated overnight with primary antibodies against ITGA8 (Santa Cruz Biotechnology, Dallas, TX, United States, #sc-365798). After primary antibody incubation, cells were washed with PBST and incubated with Alexa Fluor-594 conjugated anti-mouse secondary antibody (ThermoFisher Scientific, Waltham, MA, United States, #A21203) for 1 h at room temperature in the dark. Nuclei were stained with DAPI (1.5 μg/ml; Sigma-Aldrich, Burlington, MA, United States, #D9542). An Olympus IX51 microscope attached to Olympus DP74 camera was used to capture Images. The expression of ITGA8 was quantified by using ImageJ software. To measure the expression of the protein of interest “RawIntDen,” which represents the “Fluorescent intensity” were measured for the whole image, followed by counting the number of cells by counting the nuclei (DAPI) in the same image. Expression of the target protein was represented as the average of fluorescent intensity/number of cells of multiple fields. Triplicate experiments were performed for statistical analysis.

For immunofluorescence imaging of tissue sections, a similar protocol was performed. First, the tissue sections were deparaffinized, using xylene and grades of ethanol, followed by antigen retrieval using citrate-based Antigen Unmasking solution (Vector Laboratories, Newark, CA, United States, #H-3300) following the manufacturer’s protocol. After antigen retrieval, non-specific immunoreactions were blocked by treating the tissue sections with 1% bovine serum albumin (BSA) in PBST for 1 h. For ITGA8, the primary antibody (1:50; Santa Cruz Biotechnology, Dallas, TX, United States, #sc-365798) was incubated overnight at 4^°^C, followed by washing with PBST. Sections were incubated with anti-mouse secondary antibody conjugated to Alexa Fluor 594 (1:200 and 1:200; ThermoFisher Scientific, Waltham, MA, United States, #A21203) for 1 h at room temperature. VECTASHIELD^®^ HardSet™ Antifade Mounting Medium with DAPI (Vector Laboratories, Newark, CA, United States, #H-1500) was used for nuclei staining. An Olympus BX51 microscope attached to an Olympus DP74 camera was used for imaging.

For immunohistochemistry (IHC), tissue sections were deparaffinized in xylene and rehydrated with ethanol and PBS before antigen retrieval. Sections were incubated with 3% H_2_O_2_ at room temperature for 10 min and blocked with 2% normal goat serum (Vector Laboratories, Newark, CA, United States, #S-1,000) in TBST (Tris-buffered saline containing 0.1% Tween-20) for 1 h. After blocking, sections were incubated with primary antibody for VCL (1:50, Santa Cruz Biotechnology, Dallas, TX, United States, #sc-73614) overnight at 4^°^C. Following this, sections were incubated with SignalStain Boost anti-Mouse IHC Detection Reagent (Cell Signaling Technology, #8114) at room temperature for 45 min. Visualization was performed using SignalStain DAB Substrate Kit (Cell Signaling Technology, Danvers, MA, #8059) and imaged using a Keyence BZ-X800 Fluorescence microscope.

### Western blot analysis

Cell lysates were prepared from cultured cells in RIPA Lysis and Extraction Buffer (ThermoFisher Scientific, Waltham, MA, United States, #89900) supplemented with Halt Protease Inhibitor Cocktail (ThermoFisher Scientific, Waltham, MA, United States, #87785). Lysates were centrifuged at 15,871 g for 15 min at 4^°^C and the supernatants were collected. Pierce BCA Protein Assay Kit (ThermoFisher Scientific, Waltham, MA, United States, #23227) was used to estimate protein concentration. Approximately 10–25 μg of the cell lysates were mixed with 6X Laemmli SDS-Sample Buffer (Boston Bioproducts, Milford, MA, United States, #BP-111R) containing β-mercaptoethanol and boiled for 5 min. Protein samples were separated in 4–20% Mini-PROTEAN^®^ TGX™ Precast Gels (Bio-Rad, Hercules, CA, United States, #4561094), transferred into a polyvinylidene difluoride membrane (Bio-Rad, Hercules, CA, United States, #1620177), and blocked with 5% non-fat milk in TBS containing 0.1% Tween^®^-20. Membranes were probed with primary antibodies against ITGA8 (1:500; Santa Cruz Biotechnology, Dallas, TX, United States, #sc-365798), VCL (1:500; Santa Cruz Biotechnology, Dallas, TX, United States, #sc-25336), SMA (1:200; Abcam# ab18147), VIM (1:1,000; Cell Signaling Technology# 5741S), and GAPDH (1:1,000; Novus Biologicals, Centennial, CO, United States, #NB300-221). After the primary antibodies probing, membranes were further probed with anti-rabbit and anti-mouse secondary antibodies conjugated with HRP (1:4,000; Vector Laboratories, Newark, CA, United States, #PI-1000, PI-2000). After probing with the secondary antibody, Pierce ECL Western Blotting Substrate (ThermoFisher Scientific, Waltham, MA, United States, #32106) or SuperSignal West Dura Extended Duration Substrate (ThermoFisher Scientific, Waltham, MA, United States, #34075) was used to develop western blots. Restore™ Western Blot Stripping Buffer (Thermo Scientific# 21059) was used for re-probing with different primary antibodies following the manufacturer’s protocol. Protein levels were quantified by densitometric analysis using ImageJ software and normalized to GAPDH.

### Statistics

All the experiments were performed at least in triplicate. Mann Whitney test was performed to compare two groups and one-way analysis of variance (ANOVA) was performed to compare more than two groups to determine statistical significance using the GraphPad Prism 8 software package. For all analyses, *p* ≤ 0.05 were considered statistically significant.

### Study approval

This study was approved by the Institutional Animal Care and Use Committee at NCH (protocol AR16-00053) and conducted per the NIH’s Guide for the Care and Use of Laboratory Animals (41).

## Results

### Nitric oxide transcriptionally regulates key signaling pathways for calcification in porcine aortic valve interstitial cells

**FIGURE 1:**
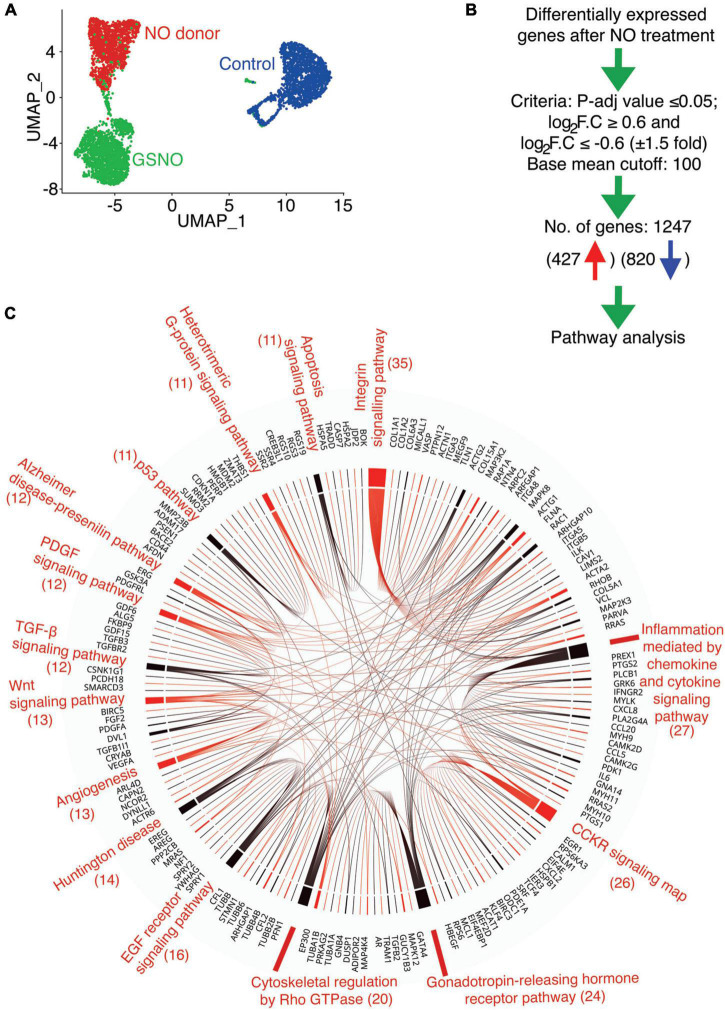
Identification of altered signaling pathway in porcine aortic valve interstitial cells (pAVICs) exposed to nitric oxide (NO) donor or S-nitrosoglutathione (GSNO) by single-cell RNA sequencing (scRNA-seq). **(A)** Uniform Manifold Approximation and Projection (UMAP) plot of scRNA-seq expression data displays three distinct populations of pAVICs cultured in the absence (control; blue) and presence of NO donor (red) or GSNO (green) for 5 days. **(B)** The flow chart represents the filtering criteria for the selection of differentially expressed genes. **(C)** Chord plot illustrates overrepresented signaling pathways (at least > 10 genes) and corresponding differentially expressed genes identified by PANTHER pathway analysis. The number of genes is mentioned in parentheses identified in each pathway.

We previously reported that pAVICs calcify spontaneously when cultured on a stiff surface. NO and GSNO can prevent this spontaneous calcification by inhibiting the activation of myofibroblast genes. To further confirm our observation, we cultured pAVICs in osteogenic media for 5 days in the presence and absence of NO donor and GSNO. We observed calcification of pAVICs in osteogenic media can also be inhibited by NO donor and GSNO (Supplementary Figure 1). Similar to this observation, we previously reported that NO reduces the protein expression of an osteogenic marker, RUNX2 in ratAVICs, cultured in osteogenic media (7). These observations in different *in vitro* calcific conditions strengthen our hypothesis that NO can inhibit valve calcification. To evaluate the NO or S-nitrosylation-dependent transcriptional regulation of cultured pAVICs, scRNAseq analysis of NO donor-treated, GSNO-treated, and untreated pAVICs was performed (7). Since, heart valves are composed of different cell types including subpopulations of VECs, VICs, immune cells, and melanocytes, bulk RNAseq cannot differentiate the cellular response to NO donor and GSNO (39, 42). However, we did not observe VECs or immune cells, or melanocytes and any separate identity of VIC-subpopulations in our pAVICs culture (7). Our transcriptomic analysis revealed activation of several myofibroblast genes in absence of NO donor and GSNO, among which *ACTA2* (αSMA) was found to be the most significantly upregulated gene (7). However, this analysis was limited to the identification of myofibroblast gene activation. In this report, we have analyzed the scRNAseq data to identify altered cellular signaling pathways that are responsive to NO. Unbiased clustering of the gene expression from all of these cells using Uniform Manifold Approximation and Projection (UMAP) demonstrated three distinct populations (Figure 1A). NO donor and GSNO treated pAVIC populations were similar in their cellular transcriptomic profiles but distinctly separate from the untreated control pAVICs. This data suggests that the NO-dependent transcriptional regulation of pAVICs is predominantly *via* S-nitrosylation (Figure 1A) based on the chemical and biological aspects of GSNO, which is considered an S-nitrosylating agent (43). Since NO exerts its cellular activity to a greater extent *via* S-nitrosylation, the effects of GSNO and NO donors are overlapping (Figure 1A). In addition, we previously demonstrated that GSNO produces an insignificant amount of NO in pAVICs culture (7). This observation also reduces the possibility of NO-dependent tyrosine nitration, another protein modification that is primarily associated with the oxidative stress-dependent generation of peroxynitrite, a highly reactive nitrogen species (RNS) (44).

To investigate the transcriptional changes involved in calcification using this *in vitro* system, we evaluated common differentially expressed genes after NO and GSNO treatment compared to untreated control with *p*_adjusted_ ≤ 0.05 and Log_2_FC ≥ 0.6 or ≤–0.6 (at least 50% increase or decrease in fold change). We considered differentially expressed genes after filtering the expression with a base mean cut-off of 100 for all of the samples. Using these filter criteria, we obtained 1,247 transcripts for protein-coding genes, among them 427 were upregulated and 820 were downregulated (Figure 1B and Supplementary Data 1). PANTHER pathway analysis of these 1,247 genes identified 15 pathways with at least > 10 genes (Figure 1C). These pathways include integrin signaling (*ITGA8, VCL, COL1A1, COL1A2, ACTA2, ACTN1, ACTG1*) with the maximum number of affected genes, followed by Rho GTPase, Wnt (*DVL1*), TGF-β (*TGFB2, TGFB3, TGFBR2*), and p53 signaling pathways. Interestingly, all of these pathways have been identified to be involved in the initiation and progression of calcification and this data suggests that these pathways are regulated by NO in the setting of CAVD (45–47). NO-dependent regulation of the integrin signaling pathway that regulates ECM by modulating actin cytoskeleton and focal adhesions has already been demonstrated in cancer and inflammatory conditions (48–51). The formation of the actin cytoskeleton and focal adhesions are also regulated by Rho family GTPases, which were also identified in the PANTHER analysis (Figure 1C). Overall, this data indicates that NO and S-nitrosylation can modulate multiple cellular signaling pathways and supports the utility of cultured pAVICs as an *in vitro* model of valve calcification.

### Nitric oxide and S-nitrosoglutathione alters the integrin signaling pathway in porcine aortic valve interstitial cells

**FIGURE 2:**
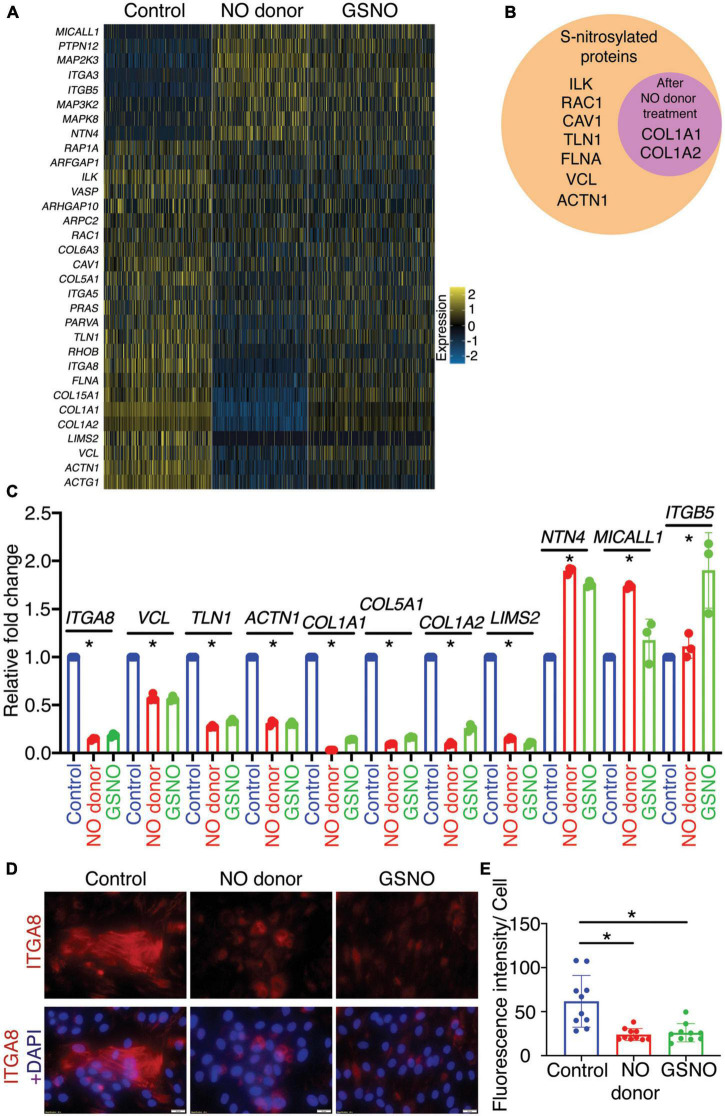
Identification and validation of differentially expressed genes involved in integrin signaling pathway in porcine aortic valve interstitial cells (pAVICs) exposed to nitric oxide (NO) donor or S-nitrosoglutathione (GSNO). **(A)** Heatmap represents *Z*-scored expression of 32 differentially expressed genes involved in the integrin signaling pathway in individual cells from control, NO donor, and GSNO treated pAVICs. **(B)** Diagram representing the protein product of differentially regulated integrin pathway genes, which were S-nitrosylated in valve interstitial cells (VICs). The circle inside represents S-nitrosylated proteins enriched after NO donor treatment. **(C)** Transcriptional expression of 11 representative genes from the integrin signaling pathway was estimated in control, NO donor, and GSNO-treated pAVICs by quantitative real time polymerase chain reaction (RT-PCR). **(D)** Immunofluorescence images represent the decreased expression of ITGA8 (red) with NO donor and GSNO when compared to untreated control pAVICs, co-stained with nuclear DAPI (blue). Scale bar: 20 μm. **(E)** The graph shows the quantification of fluorescence intensity of ITGA8 expression in control, NO donor, and GSNO treated pAVICs normalized to the number of cells. *Indicates *p* ≤ 0.05.

In addition to PANTHER pathway analysis, we utilized WebGestalt (WEB-based GEne SeT AnaLysis Toolkit) to evaluate overrepresented pathways based on their false discovery rate (FDR) (Supplementary Figures 2A,B; 52). Interestingly we observed that the Integrin signaling pathway was the top significantly enriched pathway with eight upregulated and 24 downregulated genes after NO donor and GSNO treatment as shown in the heatmap (Figure 2A). The altered gene expression pattern between the NO donor treated and untreated control was striking while the gene expression changes after GSNO treatment demonstrated an “intermediate” pattern between these two conditions. As both NO donor and GSNO equally prevented calcification *in vitro* (7), this suggested that a partial rescue of the gene expression profile was sufficient to inhibit calcification. In addition to the integrin signaling pathway, the Rho GTPase pathway was also discovered as a significantly altered pathway (Supplementary Figure 2B). As described earlier, the Rho GTPase pathway functions together with the integrin signaling pathway to modulate the response of VICs to altered ECM and vice versa in CAVD (45). Notably, among these 32 differentially expressed genes in the integrin signaling pathway, the protein products of nine genes were identified as S-nitrosylated in VICs either calcific or non-calcific conditions after NO treatment (Figure 2B; 7).

To validate the involvement of the integrin signaling pathway, we evaluated the expression of three out of eight upregulated and eight out of 24 downregulated genes. These genes represent at least one-third of the identified genes of the integrin signaling pathway using quantitative RT-PCR. Expression of these genes demonstrated similar and significant transcriptional expression as observed by scRNAseq (Figures 2A,C). These genes encode for ECM proteins (e.g., *COL1A1, COL1A2*: collagens), focal adhesion proteins (e.g., *TLN1*: talin, *VCL*: vinculin), and also integrins (*ITGA5, ITGA8, ITGB5*: integrins α and β subunits). The heterodimer of integrin β5 (*ITGB5*) and αv (integrin αvβ5) are expressed in fibroblasts and endothelial cells, whereas integrin α8 (*ITGA8*) and β1 (integrin α8β1) are expressed in smooth muscle cells (SMC) (24). The role of ITGA8 has been linked to aortic calcification in a murine model of atherosclerosis (53). Therefore, we were particularly interested in *ITGA8* expression in this study as a critical gene altered by NO. We examined the expression of Integrin α8 (ITGA8) in cultured pAVICs using immunofluorescence (Figures 2D,E). We observed reduced expression of ITGA8 in pAVICs after NO donor and GSNO exposure compared to untreated control, following a similar trend observed in our scRNAseq data. Interestingly, we observed upregulation of *ITGB5* and downregulation of *ITGA8* after NO donor and GSNO treatment suggesting that NO and GSNO inhibit the expression of SMC genes in VICs and allow VICs to maintain a more, quiescent fibroblast-like phenotype (Figure 2A). Overall, our data suggest that NO and S-nitrosylation-mediated modulation of the integrin signaling pathway may be associated with the progression of CAVD.

### Expression of integrin signaling pathway members was perturbed in the murine calcific aortic valve

**FIGURE 3:**
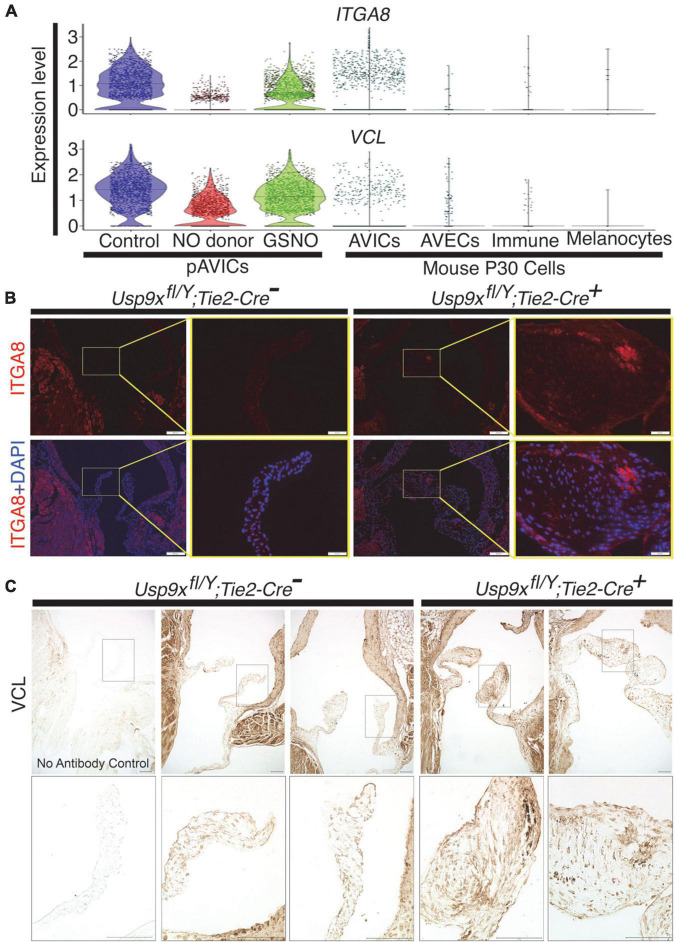
Increased expression of ITGA8 and vinculin (VCL) in calcified mouse aortic valve. **(A)** Violin plots demonstrate the expression of *ITGA8* and *VCL* in porcine aortic valve interstitial cells (pAVICs) and mouse P30 aortic valve cells. **(B)** Immunofluorescence staining of aortic valve sections demonstrates the increase of ITGA8 (Integrin α8; red) expression in calcific conditions (*Usp9x*^fl/Y^*; Tie2-Cre^+^*) compared to (*Usp9x*^fl/Y^*; Tie2-Cre^–^*) controls (*n* = 2) and co-stained with nuclear DAPI (blue). Arrows indicate the representative area of expression of ITGA8. Yellow boxes indicate the magnified area of the tissue sections shown right of each image. Scale bar: 100 μm. **(C)** Immunohistochemistry of aortic valve sections demonstrate increased expression of VCL in (*Usp9x*^fl/Y^*; Tie2-Cre^+^*) compared to (*Usp9x*^fl/Y^*; Tie2-Cre^–^*) controls (*n* = 2). Negative control with no primary antibody is shown in the first column. Boxes indicate the magnified area of tissue sections shown below each image. Scale bar: 100 μm.

As discussed above, a heterodimeric combination of integrins receptors determines ECM binding specificity and downstream signal transduction. Integrins recruit focal adhesion proteins, such as VCL that mechanically connect the ECM and cytoskeleton, to balance a force between cellular stress fibers and ECM (32). The role of VCL has previously been reported in both valvular and vascular endothelial morphological alignment (54). Endothelial cells sense the hemodynamics in their environment and contribute to calcification by responding to the shear stress experienced on the cells’ apical side (55). To test the expression of these integrin pathway genes, we combined our data with previously published scRNAseq data of mouse adult aortic valves at P30 as described previously (7, 39). We did not observe significant expression of *ITGA8* and *VCL* in healthy aortic VICs in wild-type mice (Figure 3A). However, both of these genes were highly expressed in pAVICs, cultured on the stiff surface, and downregulated after treatment with NO donor and GSNO (Figure 2C). To test whether this increase in integrin pathway genes is specific to pAVICs *in vitro* culture condition or it has any *in vivo* relevance, we utilized our newly described CAVD murine model in which *Usp9x* was deleted in endothelial and endothelial-derived cells (7). Since *Usp9x* is located in X-chromosome, therefore conditional deletion of the gene from endothelial and endothelial-derived VICs will be homozygous in male (*Usp9x*^fl/Y^*; Tie2*^Cre+^**), and heterozygous in female (*Usp9x*^fl/Wt^*; Tie2*^Cre+^**) mice. Aortic velocity was measured in female mice by echocardiography, and we observed incomplete penetrance of a stenotic phenotype in these 24 weeks old mice (*p* = 0.4498) (Supplementary Figure 3). Therefore, *Usp9x*^fl/Wt^*; Tie2*^Cre+^** female mice were not used in this study as a model of calcification. Notably in humans, males also develop calcification more often than females (56, 57). We examined the expression of ITGA8 and VCL in the aortic valve of *Usp9x*^fl/Y^*; Tie2Cre^+^* murine model in comparison to *Cre*^–^ control (Figures 3B,C). Immunofluorescence imaging demonstrated increased expression of ITGA8 in calcific valves (Figure 3B). Immunohistochemistry also demonstrated increased VCL expression in the aortic valve of *Usp9x*^fl/Y^*; Tie2Cre^+^* mice (Figure 3C). Interestingly, higher expression of ITGA8 was observed in the myocardium of *Usp9x*^fl/Y^*, Tie2Cre^–^*, compared to *Usp9x*^fl/Y^*, Tie2Cre^+^* mice (Supplementary Figure 4). This data indicates that elevated expression of ITGA8 is valve specific in response to calcification signaling events. Therefore, both our *in vivo* and *in vitro* observation demonstrates altered expression of integrin signaling pathway members, which suggests perturbed cell-ECM communication during CAVD.

### Nitric oxide-dependent integrin pathway regulation is transient

**FIGURE 4:**
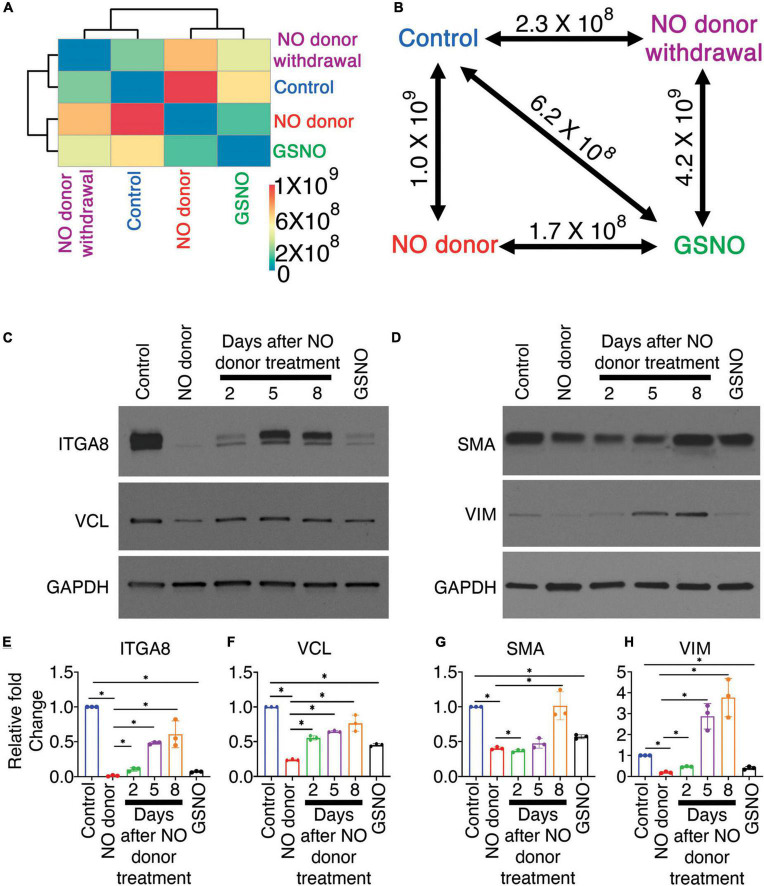
Nitric oxide (NO)-dependent transcriptional responses in porcine aortic valve interstitial cells (pAVICs) are short-lived. **(A)** Heatmap showing the Spearman distance between different pairs of culture conditions calculated based on the normalized average expression of 1,247 selected genes of pAVICs, cultured for 5 days in the absence (control) and presence of NO donor or S-nitrosoglutathione (GSNO) or a culture condition where NO donor was present for first 3 days and absent for remaining 2 days (NC). **(B)** Diagram represents the Spearman distance between groups and is denoted as Spearman distance (SD), comparing any two-culture condition marked by a double-headed arrow. **(C,D)** Immunoblots show expressions of ITGA8 (Integrin α8), VCL (vinculin), SMA (smooth muscle actin), and VIM (vimentin) in pAVICs, cultured for 5 days in the absence and presence of NO donor, GSNO, and without NO donor after 3 days of NO donor treatment. After 3 days of NO donor treatment, pAVICs were cultured for additional 2, 5, and 8 days without NO donor. Glyceraldehyde 3-phosphate dehydrogenase (GAPDH) was utilized as a loading control. **(E–H)** Graphs show quantification of ITGA8, VCL, SMA, and VIM, respectively, from immunoblots shown in panel **(C,D)**. *Indicates *P* ≤ 0.05.

Our data suggest that the effect of NO on the cellular signaling pathway is dependent on the NO-mediated post-translational modification, S-nitrosylation. S-nitrosylation is a reversible protein modification and can initiate or inhibit cellular signaling processes. To test whether the effect of NO on these signaling pathways is long-lasting, pAVICs were cultured for 3 days in presence of a NO donor, followed by culturing cells without a NO donor for two more days. Hereafter, this pAVICs culture condition will be designated as “NO donor withdrawal.” The scRNAseq data after NO donor withdrawal was analyzed in comparison to the transcriptomic data in the presence and absence of NO donor and GSNO for 5 days. In this study, we considered 1,247 identified protein-coding genes based on *P*-value and fold changes (Figure 1). We examined the correlative distance between each culture condition and observed the expression pattern of these 1,247 genes after NO donor withdrawal was similar to untreated control [Spearman distance (SD) = 2.3 × 10^8^] (Figures 4A,B). Not surprisingly, NO donor and GSNO treatment show similar expression profiles (SD = 1.78 × 10^8^). This data again is consistent with the finding that the NO-dependent transcriptional regulation in pAVICs is predominantly exerted by S-nitrosylation and this effect is transient.

To validate this observation in protein level, we cultured pAVICs for 3 days in presence of NO donor and then without NO donor for additional 2, 5, and 8 days. We examined the protein expression of ITGA8 and VCL by western blot after NO donor withdrawal in comparison to untreated control, NO donor, and GSNO-treated pAVICs (Figure 4C). Both ITGA8 and VCL protein expression was reduced after NO donor and GSNO treatment but increased again after NO donor withdrawal (Figures 4C,E,F). Interestingly, the restoration of the expression profile of a gene after NO donor withdrawal appeared to be slower at the translational level compared to the transcriptional level (Figures 4A,C). This is possibly due to the reversibility of S-nitrosylation modification, which is removed slowly from the target proteins in the absence of NO. This observation indicates that the effect of NO and subsequent S-nitrosylation on keeping VICs healthy is a temporary event. Continuous NO exposure, either from endogenous or external sources is necessary to keep VICs healthy and quiescent fibroblast behavior.

We previously demonstrated that pAVICs undergo myofibroblast activation spontaneously and NO can inhibit this activation by downregulation of *ACTA2* (SMA: smooth muscle actin) and *VIM* (Vimentin) expression at the transcriptional level (7). Here, we found the protein expression of SMA and VIM were also reduced after NO donor and GSNO treatment compared to untreated control. Similar to integrin pathway members, SMA and VIM expression was also increased after NO donor withdrawal (Figures 4D,G,H). SMA and VIM are both structural proteins important for generating forces, which are transmitted to the ECM *via* integrins and focal adhesion proteins. NO, and GSNO can inhibit the altered expression of ECM remodeling genes, both at the transcriptional and translational levels.

## Discussion

In this study, we evaluated the transcriptional changes of cultured pAVICs in the presence and absence of NO donor and GSNO. Differentially expressed genes from various signaling pathways were identified in presence of NO donor and GSNO compared to untreated control. We considered 1,247 significantly (*P*_adjusted_ ≤ 0.05) expressed genes with at least 1.5-fold up or down-regulation for pathway enrichment analysis. Our analysis revealed the involvement of integrin, Rho GTPase, Wnt, TGF-β, and p53 signaling pathways in the process of calcification. We detected the highest number of genes involved in the integrin signaling pathway were differentially expressed after NO donor and GSNO treatments. We verified the expression of integrin pathway members and their temporal regulation by NO donor utilizing both pAVICs culture and a murine model of calcification. Overall, our study suggests that NO regulates the cell-ECM interaction by modulating the integrin pathway, which subsequently leads to calcification *via* dysregulated cellular signaling.

With analysis of our scRNAseq data from cultured pAVICs exposed to NO donor and GSNO, we identified eight upregulated and 24 downregulated genes that encode for important components of this signaling pathway, including different integrins, collagens, and focal adhesion proteins (Figure 1C). Interestingly, the expression of these genes after GSNO treatment was in between NO donor treated and untreated control (Figure 2A). This observation generates a possibility of involvement of molecular pathways other than S-nitrosylation. It is important to note that we previously demonstrated that the NO-dependent sGC/cGMP pathway is not activated in pAVICs after NO donor treatment (7). Possibly the differences in gene expression profile between NO donors and GSNO are due to differential redox status, which was not investigated and is beyond the scope of this study. In addition to S-nitrosylation, another NO-dependent post-translational modification is tyrosine nitration, which also plays an important role in regulating protein structure and activity (58–60). Nitration of tyrosine residues is classically produced by peroxynitrite, generated by the reaction of NO and oxygen radicals, associated with oxidative stress. Traditionally, it was thought to be an irreversible, degenerative process that leads to protein degradation (61–63). However, there is accumulating evidence demonstrating its reversible nature in certain conditions, qualifying this modification as an inducer of cellular signaling, similar to phosphorylation-dephosphorylation or S-nitrosylation- denitrosylation (58–60). The functional role of tyrosine nitration in the context of calcification needs to be studied further. On the other hand, S-nitrosylation is an established redox-dependent reversible protein modification, important for various cellular signaling. However, aberrant production of ROS and RNS leads to alteration in protein folding and function *via* dysregulated S-nitrosylation and tyrosine nitration. Dysregulation of these protein modifications is already implicated in aging, cancer, diabetes, and also neurodegenerative, cardiovascular, pulmonary and musculoskeletal diseases (64, 65). Therefore, a regulated balance of NO production and cellular redox status is required to propagate healthy cell-ECM interaction.

We verified similar changes in the expression of integrins (e.g., ITGA8) and focal adhesion proteins (e.g., VCL) in the murine calcified aortic valve, as observed in scRNAseq (Figures 2, 3). Previously we described thickened and stenotic aortic valve with a disorganized matrix in *Usp9x*^fl/fl^*; Tie2-Cre^+^* mouse (7). It is well-known that the production of NO by endothelial NOS is dependent on blood flow (12, 13). It can be speculated that thickened valve leaflets would initiate disturbed blood flow patterns across the valve, which would also lead to perturbed NO production. Since NO is a freely diffusible molecule, but short-lived, it cannot travel from the endothelial cells to the interstitial cells located far (aortic side) from the laminar shear flow side (ventricular side) in the thickened valve. It is notable that the aortic side of the aortic valve experience oscillatory shear flow (66). We previously demonstrated that S-nitrosylation is predominantly present in the ventricular side of the aortic valve and the atrial side of the mitral valve, both of which experience the laminar shear flow (7). Therefore, we suggest that the perturbed blood flow through the thickened and stenotic aortic valve in our murine model of CAVD leads to insufficient NO production, followed by perturbation of the integrin signaling pathway and ECM turnover in VICs.

Integrins demonstrate binding specificity toward various ECM proteins and transduce intracellular signals. This signaling is determined by the ECM composition and stiffness, which regulate cellular phenotypes (24). We observed differential expression of integrin genes (*ITGA3,5* and *8*) between control and NO-treated pAVICs (Figure 1C). The protein product of these three genes heterodimerizes with integrin β1. All of these integrin heterodimers α3β1, α5β1, and α8β1 play important roles in promoting myofibroblast activation and developing fibrotic conditions (67–70). In addition, we observed differential expression of various collagens (*COL1A1, COL1A2, COL15A1, COL6A3, COL5A1*) (Supplementary Figure 2A), which constitutes the largest fraction of the ECM, both in the valve and in the myocardium. After binding to ECM, integrins interact with the cytoskeleton *via* focal adhesion proteins, which were also differentially expressed (*VCL, TLN1*) after NO and GSNO treatment (Figure 1C). Interestingly, two collagens (*COL1A1, COL1A2*), which were transcriptionally downregulated (Figure 2A and Supplementary Figure 2A) were also S-nitrosylated in the non-calcific condition after NO donor treatment (Figure 2B). This data suggests a possible feedback inhibition of ECM genes at the transcriptional level by S-nitrosylation. However, the mechanism of this feedback regulation requires further investigation.

In addition, changes in the expression of the cytoskeletal genes including *ACTA2, ACTN1*, and *ACTG1* (Figure 1C) were observed after NO donor and GSNO treatment. The cytoskeletal response toward ECM is mediated by the Rho family of GTPases, which were also differentially expressed (*RHOB, RAC1*) in NO donor and GSNO-treated cells (Figure 1C). Intracellular forces generated by the actin cytoskeleton are transmitted back to the ECM to alter its organization. This triggers the activation of TGF-β, which can further promote myofibroblast activation (71). TGF-β also regulates the Wnt signaling pathway by repressing GSK3β, which results in the degradation of cytoplasmic β-catenin in cooperation with disheveled (DVL). We found differential expression of TGF-β and Wnt pathway genes (*TGFB2, TGFB3, TGFBR2, DVL1*) (Figure 1C and Supplementary Data 1), which have well-established roles in cardiac development and disease (47, 72). Overall, our scRNAseq analysis indicates that NO can globally regulate intracellular signaling pathways in VICs and its extracellular communication with ECM to maintain healthy VICs.

Healthy VICs reflect a quiescent fibroblast type which becomes activated to a myofibroblast-like state when cultured on a stiff surface of a tissue culture plate (20, 21, 73). We have demonstrated that NO donor and GSNO can inhibit the myofibroblast activation of pAVICs (7). However, it is not clear whether activated pAVICs can differentiate back to a fibroblast-like state. Interestingly, we observed increased expression of *TCF21*, a cardiac fibroblast marker after NO donor exposure (Supplementary Figure 5). It has been demonstrated before that S-nitrosylation and TCF21^+^ cells are present predominantly in the laminar shear flow side (ventricular side) of the healthy aortic valve (7, 39). This data indicates a possible correlation between NO-mediated S-nitrosylation and the maintenance of a healthy fibroblast-type state defined by TCF21 expression. An increase of *TCF21* in presence of NO (Supplementary Figure 5) also indicates that NO can reverse the myofibroblast toward a fibroblast-type state in an *in vitro* setting. Further studies are required to determine if there is a reduction of TCF21^+^ cells in the aortic valve of murine models of CAVD.

Analysis of our scRNAseq data revealed that NO-dependent regulation of the integrin signaling pathway is important for the communication between cells and ECM. However, cultured pAVICs on tissue culture plates do not have a proper ECM. Therefore, the effect of ECM remodeling during the progression of calcification cannot be identified in this *in vitro* condition, which is a major drawback of this model system. Although we verified the expression of some genes *in vivo* mouse model, the conclusion of this study is limited to the identification of altered cellular signaling pathways and gene expression, which are related to cell-ECM interaction. It has previously been reported that myofibroblast activation and calcification of pAVICs are influenced by the stiffness of the culture plates, which possibly mimics the very advanced stages of CAVD (21, 74, 75). Interestingly, NO donor and GSNO can prevent this myofibroblast activation and calcification of pAVICs, cultured on a stiff surface. This effect of NO donor and GSNO on the pAVICs is transcriptional and not influenced by the stiffness of its culture condition. This generates the possibility of therapeutic agents, which can continuously produce NO, and would have the potential to preserve the healthy status of VICs. This puts NO donors and GSNO as potential therapeutic agents which may fix or decelerate the progression of CAVD by altering diseased VICs and ECM *via* regulation of integrin and other involved signaling pathways.

**FIGURE 5:**
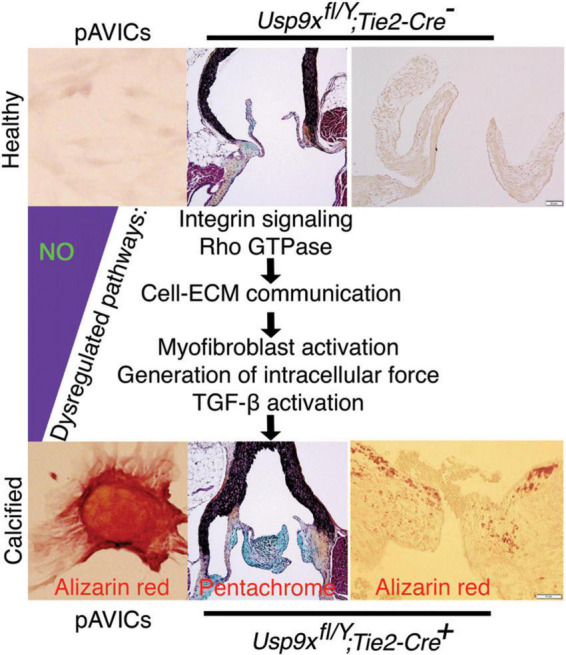
Nitric oxide (NO) inhibits the process of aortic valve calcification. Schematic showing the proposed model where calcification is initiated with reduced NO availability that perturbs integrin signaling pathway, cell-extracellular matrix (ECM) interactions, followed by dysregulation of various cellular signaling pathways.

Overall, we are proposing that uninterrupted and adequate NO production is required to inhibit CAVD by keeping VICs in a quiescent fibroblast state which regulates healthy ECM turnover (Figure 5). Insufficient bioavailability of NO leads to dysregulated integrin signaling, ECM turnover, and remodeling, which generates mechanical forces both by ECM and VICs. These mechanical forces induce dysregulation of multiple downstream cellular pathways, including the TGF-β and Wnt pathways contributing to aortic valve calcification (Figure 5).

## Data availability statement

The datasets presented in this study can be found in online repositories. The names of the repository/repositories and accession number(s) can be found below: https://www.ncbi.nlm.nih.gov/geo/, GSE161123.

## Ethics statement

The animal study was reviewed and approved by Institutional Animal Care and Use Committee (IACUC) at the Research Institute at Nationwide Children’s Hospital.

## Author contributions

VG and UM: conceived the project and designed the experiments. UM, YU, and TC: performed and analyzed the experiments. MB: performed scRNA-seq library preparation. SM: processed the scRNAseq datasets and developed Ryabhatta app. UM, TC, and VG: wrote the manuscript with input from all authors. All authors contributed to the article and approved the submitted version.

## Footnotes

1
https://www.ncbi.nlm.nih.gov/assembly/GCF_000003025.6/

2
https://useast.ensembl.org/info/data/biomart/index.html

## Supplementary material

Supplementary Figure 1 Alizarin red staining of calcific nodules of porcine aortic valve interstitial cells (pAVICs) cultured on plastic tissue culture plates in osteogenic media in the presence and absence of nitric oxide (NO) donor and S-nitrosoglutathione (GSNO).

Supplementary Figure 2 The maximum number of differentially expressed genes involved in the integrin signaling pathway in porcine aortic valve interstitial cells (pAVICs) exposed to nitric oxide (NO) donor or S-nitrosoglutathione (GSNO). **(A)** Volcano plot of log_2_(Fold change) vs. –log10(*P*-value), demonstrating differential expression of upregulated (blue) and downregulated (cyan) genes between NO donor treated and untreated pAVICs. Genes identified from integrin signaling pathway have been marked. ITGA8 is one of the most significantly downregulated gene after NO donor treatment. **(B)** Graph describes overrepresented pathway analysis considering –log_10_ (FDR) and log_2_ (enrichment ratio) using WebGestalt online tool. Integrin signaling pathway, cytoskeletal regulation of Rho GTPase and CCKR signaling pathway were significant based on –log10(FDR). Intensity in color scale (top left corner) indicates number of genes identified in each pathway.

Supplementary Figure 3 The aortic velocity of female *Usp9x*^fl/wt^*, Tie2Cre^–^*, and *Usp9x*^fl/wt^*, Tie2Cre^+^* mice by echocardiography.

Supplementary Figure 4 Higher expression of ITGA8 was observed in the myocardium of *Usp9x*^fl/Y^*, Tie2Cre^–^*, compared to *Usp9x*^fl/Y^*, Tie2Cre^+^* mice.

Supplementary Figure 5 Nitric oxide (NO) donor induces the expression of the cardiac fibroblast gene, *TCF21*, in porcine aortic valve interstitial cells (pAVICs). Violin plots show increased expression of *TCF21* in pAVICs after NO donor treatment compared to untreated control.

Supplementary Data 1 Differentially expressed genes in porcine aortic valve interstitial cells (pAVICs) after nitric oxide (NO) donor and S-nitrosoglutathione (GSNO) treatment compared to untreated cells.

Supplementary Data 2 List of primers used to identify gene expression by quantitative real time polymerase chain reaction (RT-PCR).
