# Supplementary figures and images for: Single-cell RNA-sequencing analysis of aortic valve interstitial cells demonstrates the regulation of integrin signaling by nitric oxide
Source: Front Cardiovasc Med. 2022 Oct 25;9:742850. doi: 10.3389/fcvm.2022.742850 (PMC9640371; doi:10.3389/fcvm.2022.742850)

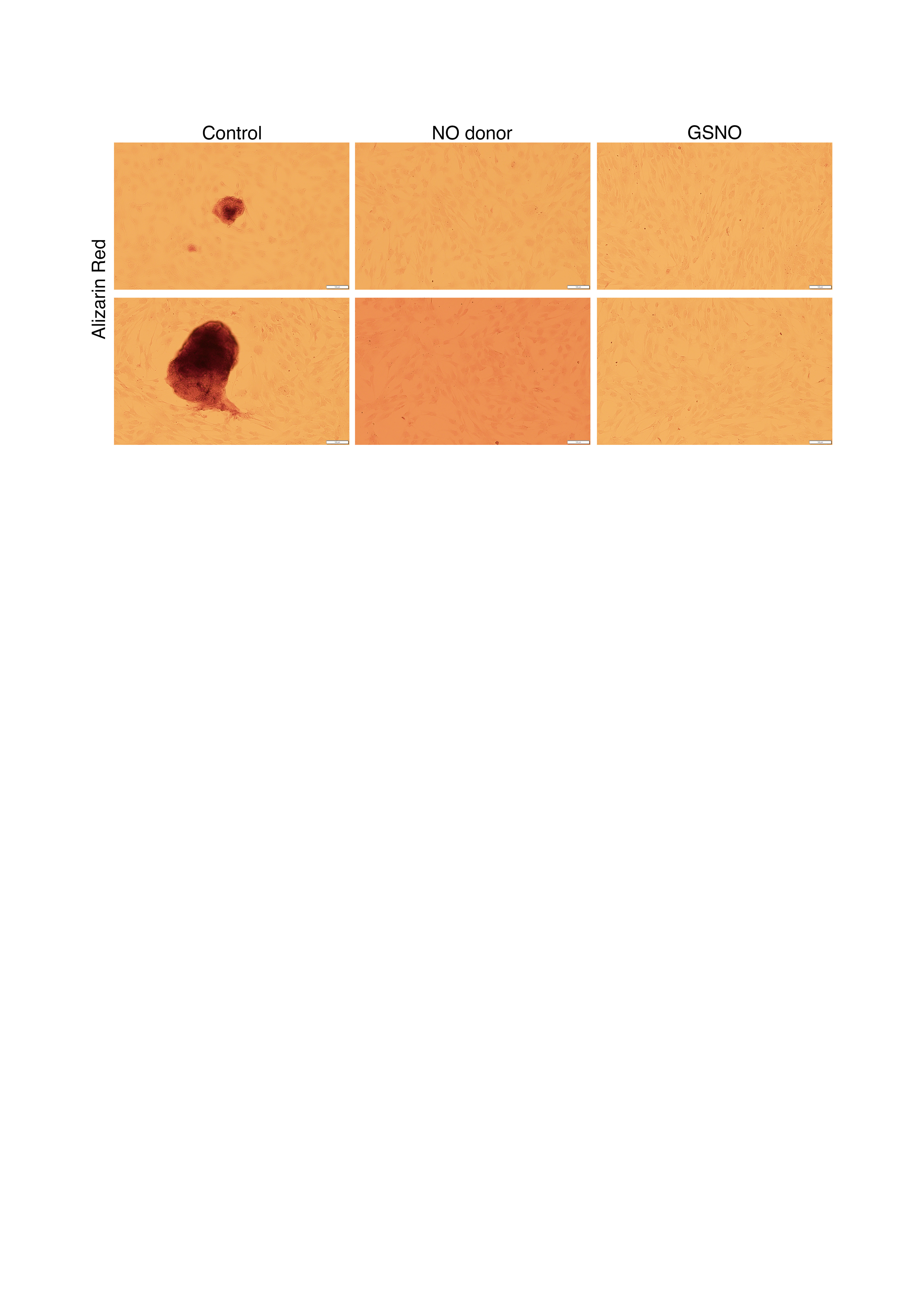

Supplement: Supplementary Figure 1 — Alizarin red staining of calcific nodules of porcine aortic valve interstitial cells (pAVICs) cultured on plastic tissue culture plates in osteogenic media in the presence and absence of nitric oxide (NO) donor and S-nitrosoglutathione (GSNO). [file Image_1.JPEG]

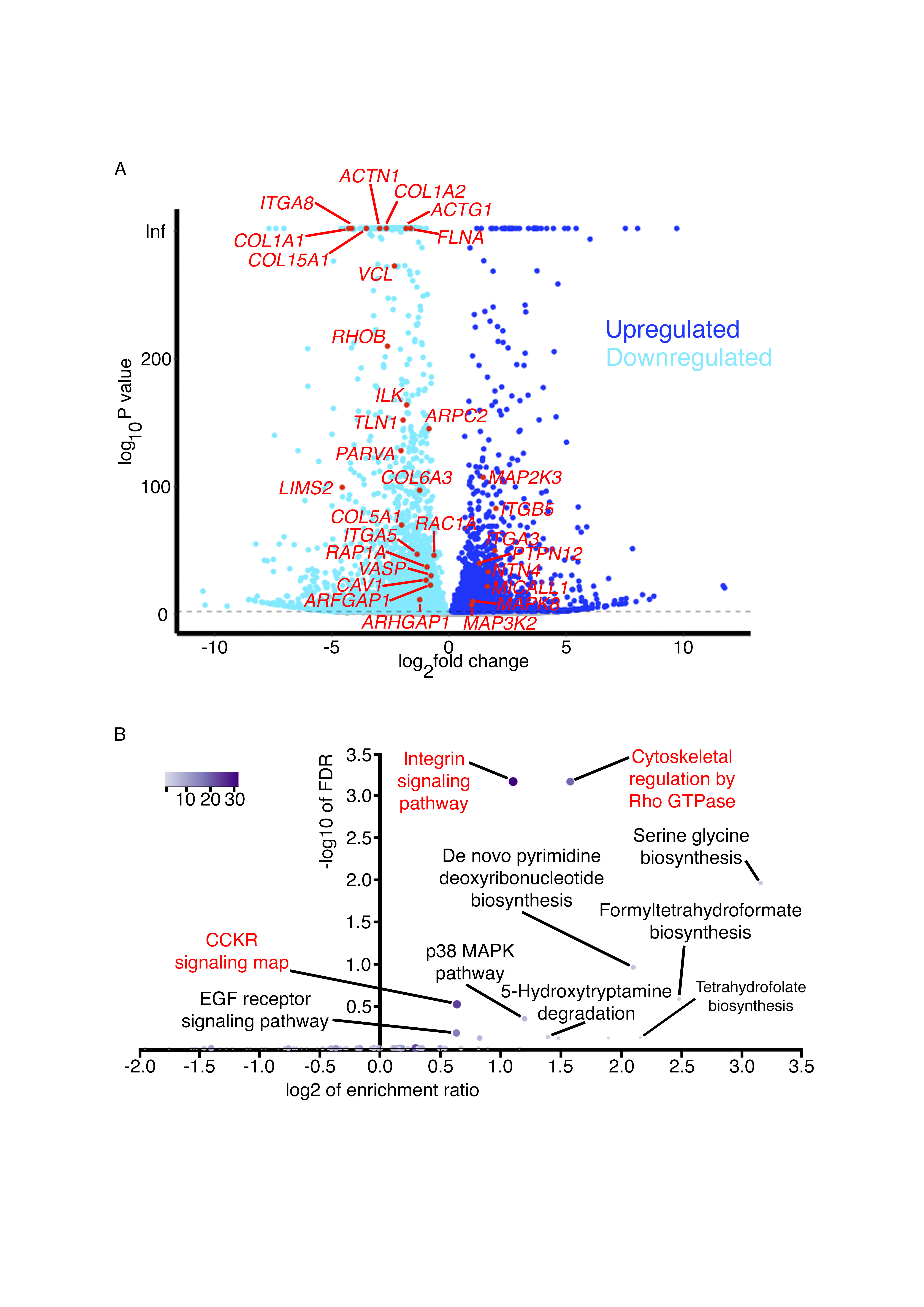

Supplement: Supplementary Figure 2 — The maximum number of differentially expressed genes involved in the integrin signaling pathway in porcine aortic valve interstitial cells (pAVICs) exposed to nitric oxide (NO) donor or S-nitrosoglutathione (GSNO). (A) Volcano plot of log2(Fold change) vs. –log10(P-value), demonstrating differential expression of upregulated (blue) and downregulated (cyan) genes between NO donor treated and untreated pAVICs. Genes identified from integrin signaling pathway have been marked. ITGA8 is one of the most significantly downregulated gene after NO donor treatment. (B) Graph describes overrepresented pathway analysis considering –log10 (FDR) and log2 (enrichment ratio) using WebGestalt online tool. Integrin signaling pathway, cytoskeletal regulation of Rho GTPase and CCKR signaling pathway were significant based on –log10(FDR). Intensity in color scale (top left corner) indicates number of genes identified in each pathway. [file Image_2.JPEG]

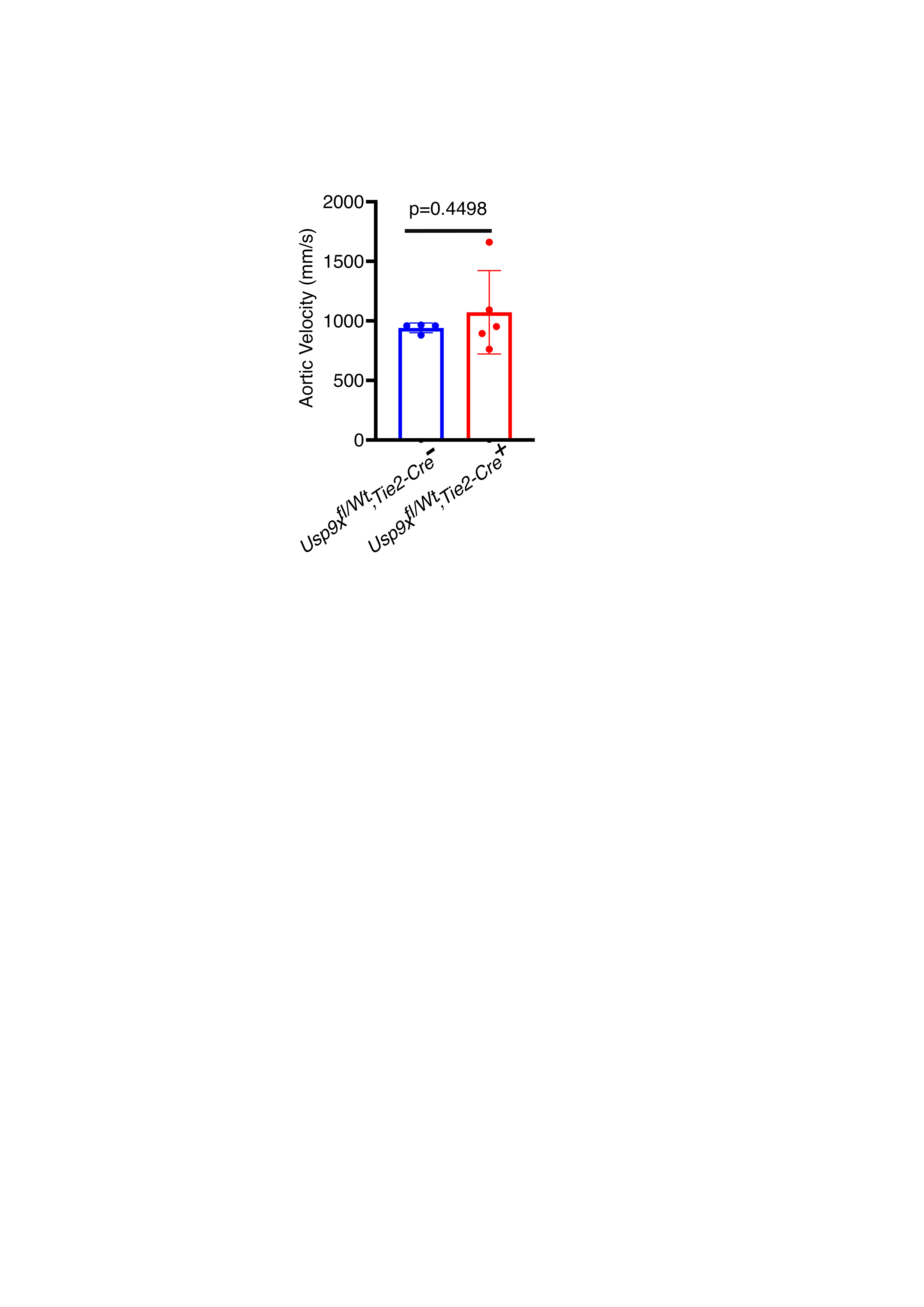

Supplement: Supplementary Figure 3 — The aortic velocity of female Usp9xfl/wt, Tie2Cre–, and Usp9xfl/wt, Tie2Cre+ mice by echocardiography. [file Image_3.JPEG]

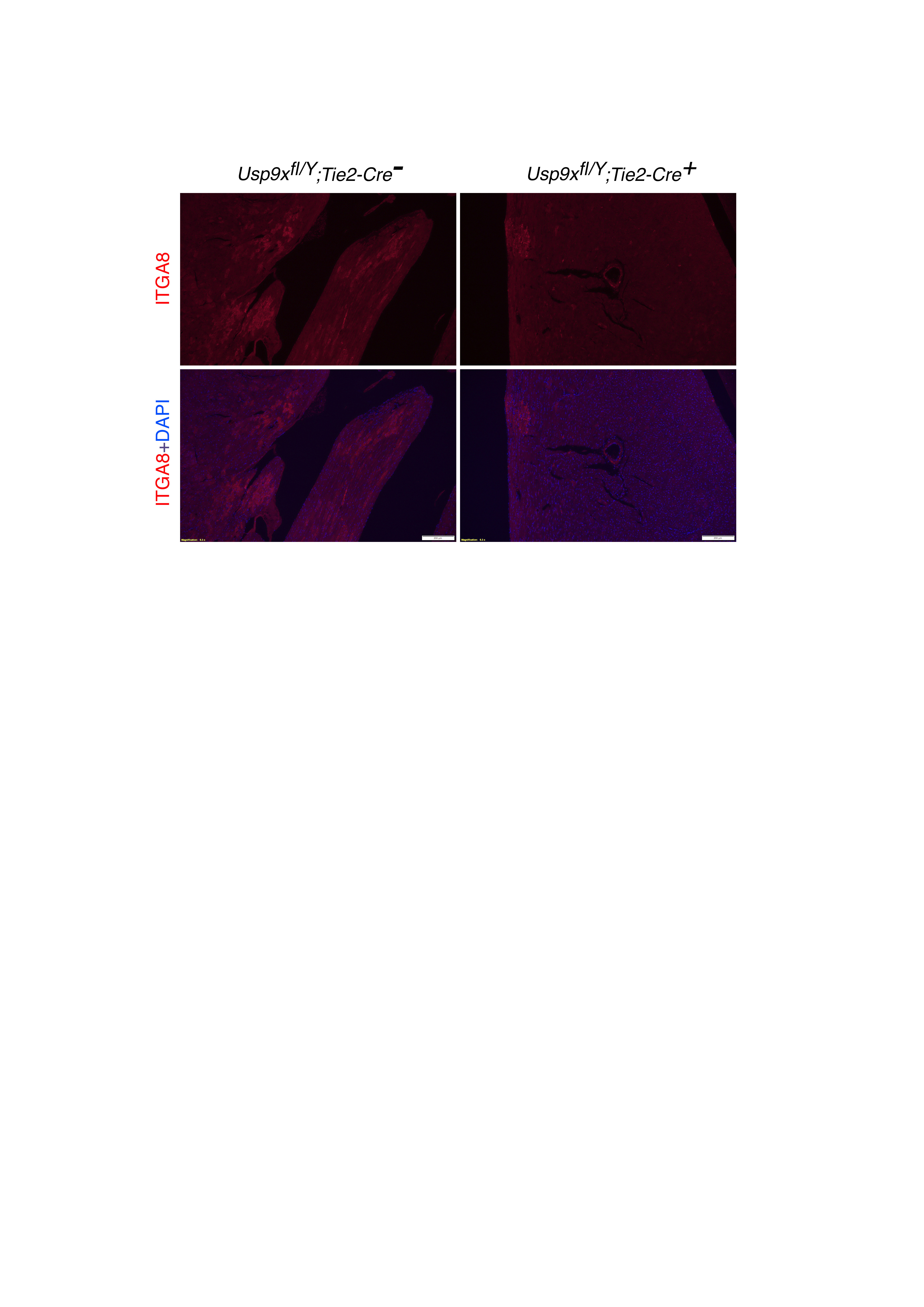

Supplement: Supplementary Figure 4 — Higher expression of ITGA8 was observed in the myocardium of Usp9xfl/Y, Tie2Cre–, compared to Usp9xfl/Y, Tie2Cre+ mice. [file Image_4.JPEG]

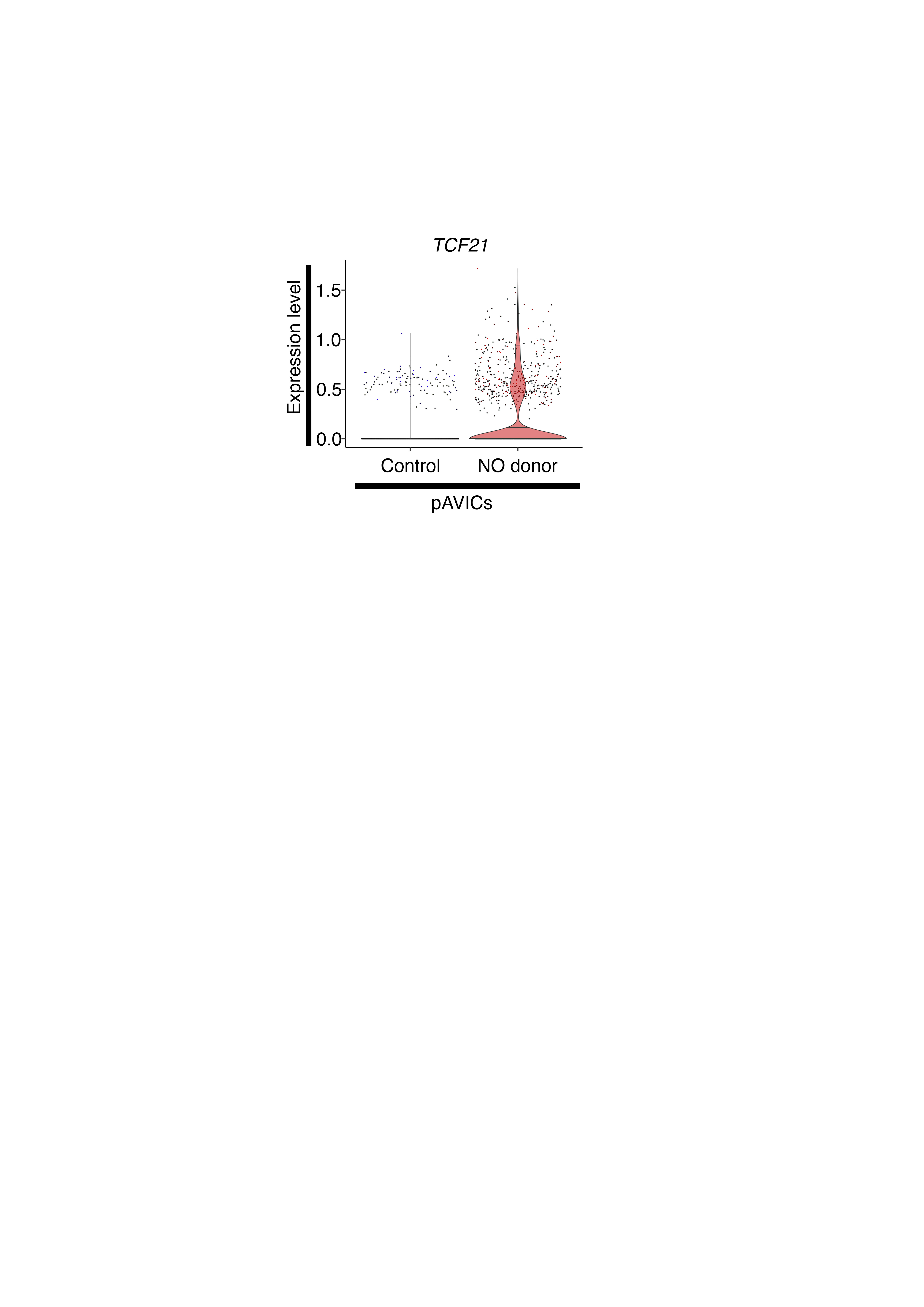

Supplement: Supplementary Figure 5 — Nitric oxide (NO) donor induces the expression of the cardiac fibroblast gene, TCF21, in porcine aortic valve interstitial cells (pAVICs). Violin plots show increased expression of TCF21 in pAVICs after NO donor treatment compared to untreated control. [file Image_5.JPEG]
